# The role of proteoglycan form of DMP1 in cranial repair

**DOI:** 10.1186/s12860-022-00443-4

**Published:** 2022-09-30

**Authors:** Yang Liu, Pingping Niu, Mengqi Zhou, Hui Xue

**Affiliations:** 1 Central Laboratory, The First Affiliated Hospital of Qiqihaer Medical University, Qiqihaer, Heilongjiang China; 2 Department of Implantology, School & Hospital of Stomatology, Tongji University, Shanghai, China; 3 Department of Endodontics, Shanghai Stomatological Hospital & Oral Biomedical Engineering LaboratoryShanghai Stomatological HospitalFudan University, Shanghai, China; 4 Department of Stomatology, The First Affiliated Hospital of Qiqihaer Medical University, 26 Xiangyang Road, Fulaerji District, Heilongjiang 161041 Qiqihaer, China

**Keywords:** Cranial defect, Glycosylation, Dentin matrix protein 1, Osteogenesis

## Abstract

**Background:**

The cranial region is a complex set of blood vessels, cartilage, nerves and soft tissues. The reconstruction of cranial defects caused by trauma, congenital defects and surgical procedures presents clinical challenges. Our previous data showed that deficiency of the proteoglycan (PG) form of dentin matrix protein 1 (DMP1-PG) could lead to abnormal cranial development. In addition, DMP1-PG was highly expressed in the cranial defect areas. The present study aimed to investigate the potential role of DMP1-PG in intramembranous ossification in cranial defect repair.

**Methods:**

Mouse cranial defect models were established by using wild- type (WT) and DMP1-PG point mutation mice. Microcomputed tomography (micro-CT) and histological staining were performed to assess the extent of repair. Immunofluorescence assays and real-time quantitative polymerase chain reaction (RT‒qPCR) were applied to detect the differentially expressed osteogenic markers. RNA sequencing was performed to probe the molecular mechanism of DMP1-PG in regulating defect healing.

**Results:**

A delayed healing process and an abnormal osteogenic capacity of primary osteoblasts were observed in DMP1-PG point mutation mice. Furthermore, impaired inflammatory signaling pathways were detected by using RNA transcription analysis of this model.

**Conclusions:**

Our data indicate that DMP1-PG is an indispensable positive regulator during cranial defect healing.

**Supplementary Information:**

The online version contains supplementary material available at 10.1186/s12860-022-00443-4.

## Background

Craniomaxillofacial bone defects severely affect the facial morphology and physiological function of patients. Craniofacial defect healing is not only a clinical issue, but also an important system for basic scientific research [1]. The regeneration of craniomaxillofacial bone defects remains a major challenge and an aggregate burden in the field of regenerative medicine after trauma, dysplasia and tumor resection [2]. To date, the known healing process of bone defect repair includes intramembranous bone regeneration and endochondral ossification [3]. Briefly, a hematoma is formed followed by vascular invasion and aggregation of mesenchymal stem cells (MSCs) at the trauma sites. Then, under the stimulation of cytokines and inflammatory factors released by immune cells, the condensed MSCs directly give rise to chondrocytes or osteoblasts, as determined by hypoxia and the mechanical environment. Finally, intramembranous ossification or endochondral ossification regenerates the typical bone structure and restores impaired bone defect repair [4].

During cranial defect healing, stem cells derived from the periosteum, bone marrow and dura migrate to the defect area and differentiate into functional osteoblasts and osteocytes secreting bone matrix and bridging injury sites. Importantly, in addition to the above significant cellular components, new results have helped elucidate the role of the extracellular matrix (ECM) in bone defect repair [5, 6]. Bone ECM, which is secreted by cells to fill the extracellular space, can interact with osteoblasts and osteoclasts to regulate ossification during the process of defect repair [7]. The ECM also provides an intricate bioenvironment with properly regulated biochemical and mechanical properties for bone injury healing. The organic ECM mainly consists of type I collagen and noncollagenous proteins. The noncollagenous proteins of the ECM can be classified into four forms: proteoglycans (PGs), glycoproteins, g-carboxyglutamate-containing proteins, and small integrin-binding ligands N-linked glycoproteins (SIBLINGs) [8]. The typical components of PGs are glycosaminoglycan (GAG) and a protein core. Although the percentage of PGs is less than 5% in bone injury sites [3], PGs play a crucial role in cell proliferation, mineral mineralization and bone remodeling during bone formation [9]. Impaired angiogenesis and bone fracture healing could be detected in mouse injury models with specific PG breakdown [10].

DMP1 is an acidic ECM and mainly regulates biomineralization in the bone matrix during the process of osteogenesis [11]. Morphological changes and bone mineral loss were observed in *Dmp1*-null mice [12]. In the bone matrix, translational modification of DMP1 undergoing proteolytic processing and phosphorylation was confirmed by protein chemistry studies [13]. Full-length DMP1 is processed into C-terminal and N-terminal segments after proteolytic modification, and the C-terminal segment is a functional fragment, that regulates bone mineralization [14]. The N-terminal segment is a special form of high molecular weight PG (named DMP1-PG), which contains a core protein and a GAG chain [15]. In mice, the GAG chain is linked to the DMP1 N-terminus at the serine 89 site, which is a highly conserved glycosylation site.

To further detect the potential function of DMP1-PG, we constructed a DMP1-PG point mutation (the serine 89 site was substituted with glycine, S89G-DMP1) mouse by disrupting the glycosylation site using a gene knock-in technique. Thinner cortical bone, decreased trabecular number and diminished osteocyte lacuna were detected in the S89G-DMP1 mice [11]. Interestingly, craniomaxillofacial deformities and cranial structure abnormalities were also clearly observed [16]. Additionally, our previous studies showed that DMP1-PG was highly expressed in the cartilage callus of fracture sites, and the S89G-DMP1 mouse fracture endochondral ossification model displayed abnormal chondrogenesis and delayed fracture healing. However, the potential role of DMP1-PG in intramembranous osteogenesis during cranial defect healing is still unknown. In the present research, we systemically investigated the function of DMP1-PG in cranial healing by employing a cranial defect mouse model. Abnormal morphology of trauma sites and downregulated expression levels of osteogenic genes were observed in the S89G- DMP1 mice. Based on our findings, we speculated that DMP1-PG could serve as a pivotal extracellular matrix proteoglycan and regulate the repair process.

## Results

### Construction of the mouse defect model

**Fig. 1:**
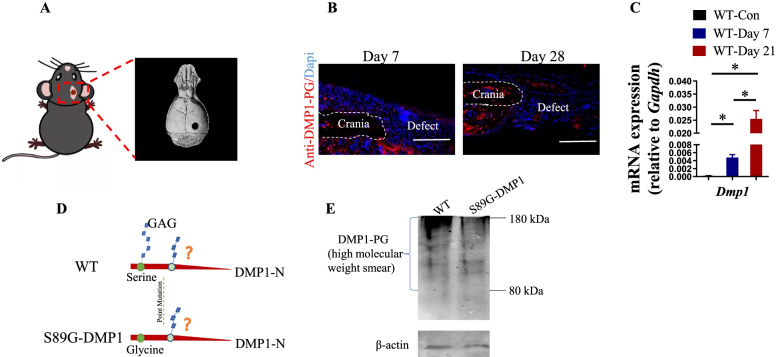
Construction of the cranial defect model. **A** Graphic representation of the cranial defect model. **B** DMP1-PG was highly expressed in the defect sites of the model mice, scale bars = 100 µm. **C** RT‒qPCR quantification of *Dmp1* in the defect sites of the WT mice. *p < 0.05, *n* = 4. **D** Strategy for the DMP1-PG point mutation in the mouse model. **E** The expression of DMP1-PG protein (high molecular weight smear) was significantly decreased in the bone matrix in the S89G-DMP1 mice

To detect the expression level of DMP1-PG in the cranial injury sites, we successfully constructed a defect model in mice (Fig. 1A). DMP1-PG was highly expressed in the defect areas and surrounding tissues, as shown by using immunofluorescence staining at days 7 and 28 post-operation in the WT group (Fig. 1B). Compared with that in the normal skull tissues, the expression level of *Dmp1*displayed a continuously increasing trend in the defect sites during defect repair (Fig. 1C). To further detect the critical role of DMP1-PG in cranial defect healing, we used the gene knock-in technique to substitute serine 89 with glycine, and a DMP1-PG point mutation mouse model was successfully constructed (Fig. 1D). After the S89G substitution, the DMP1-PG band displayed a weaker “smear” between 80 and 180 kDa in the S89G-DMP1 mouse group (Fig. 1E), indicating a small amount of residual GAG.

### Impaired osteogenesis in the defect sites of the S89G-DMP1 mice

**Fig. 2:**
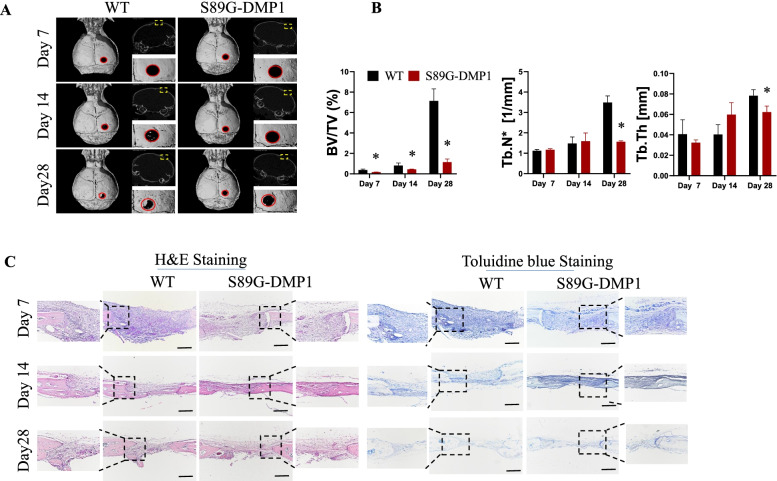
Delayed cranial defect healing in the S89G-DMP1 mice. **A** Representative micro-CT images of cranial defect sites in the WT and S89G-DMP1 groups at days 7, 14 and 28 post-injury. **B** Micro- CT measurements of BV/TV, Tb.N and Tb.Th, *p < 0.05, *n* = 3. BV/TV, new bone volume in the cranial defect sites; Tb.N, trabecular number; Tb.Th, trabecular thickness. **C** Histological analysis of cranial defect healing in the two groups. Scale bars = 500 µm

To compare the process of defect healing in the WT and S89G-DMP1 mice, we performed micro-CT scanning, and three-dimensional reconstruction revealed discrepancies in callus formation between the two groups. The newly formed bones began to bridge the cranial defect areas at day 14 in the WT mice; however, a decreased number of regenerated bone tissues was detected in the injury sites in the S89G-DMP1 mice. At day 28 post-operation, partial cortical bones covered the defect sites in the WT group, while only low levels of bones could be detected in the S89G-DMP1 mice (Fig. 2A). In addition to the 3D images of micro-CT, quantitative parameters, including newly formed bone volume/total defect volume (BV/TV), trabecular thickness (Tb.Th) and trabecular number (Tb.N) were employed, and these parameters all showed decreased trends in the S89G-DMP1 mice compared with the control mice (Fig. 2B). Histological staining showed the abnormal repair process of the defect cranial sites in the S89G-DMP1 mice. Toluidine blue is a basic heterochromatic thiazine dye, that can bind with acid groups and PGs. Therefore, toluidine blue staining can be used to observe the generation of acidic proteins and PGs in newborn bone tissue. H&E and toluidine blue staining revealed fewer osseous calluses and more fibrous calluses around the defect areas in the S89G-DMP1 mice at days 7 and 14. Moreover, the defect gaps were diminished in the WT mice, and the bony callus continuity remained poor in the S89G-DMP1 groups at day 28 post-injury (Fig. 2C).

### Lack of DMP1-PG downregulated osteogenic markers in the S89G-DMP1 mice

**Fig. 3:**
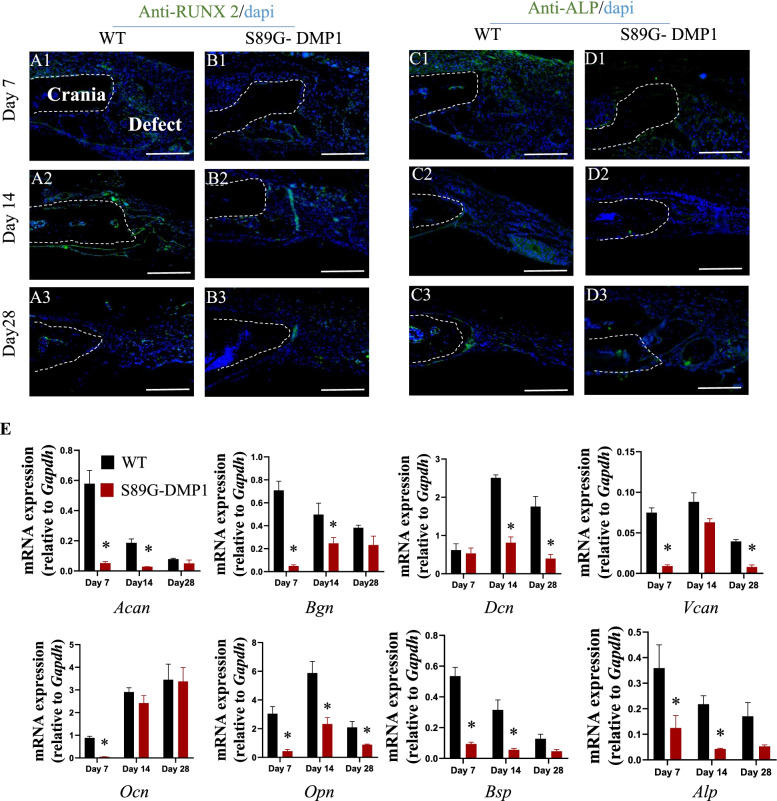
Alterations in osteogenic and PG markers in the injury sites of the WT and S89G-DMP1 mice. (A1-D3) The S89G-DMP1 mice exhibited decreased immunoreactivity of RUNX2 and ALP in the cranial defect sites. Scale bars = 100 µm. **E** RT‒qPCR analysis of osteogenic and PG-related markers. *p < 0.05, *n* = 4

To further detect the potential role of DMP1-PG in the cranial defect repair process, we used immunofluorescence staining in the control group and the S89G-DMP1 group. Downregulation of osteogenic proteins, including RUNX2 and ALP, was observed in the S89G-DMP1 mice at days 7, 14 and 28 post-operation (Fig. 3A). In addition to the immunoreactivity, we performed RT‒qPCR detection, and lower gene expression levels of PG-related and osteogenic markers were evident in the S89G-DMP1 mice during defect healing (Fig. 3E). Collectively, our results suggested that DMP1-PG might be a positive regulator of trauma repair during the process of healing.

### Abnormal osteoblast activity in vitro

**Fig. 4:**
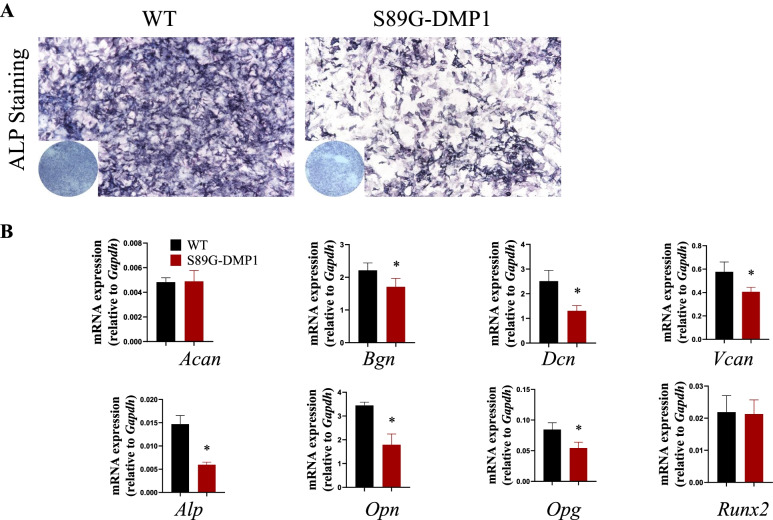
Cellular changes in the cranial defect areas of the WT and S89G-DMP1 mice. **A** Comparison of representative ALP staining of primary osteoblasts from the WT and S89G-DMP1 groups. **B** The mRNA levels of PG-related and osteogenesis-related genes were detected by RT‒qPCR. *p < 0.05, *n* = 4

Given the close relationship of osteoblasts and defect repair, cranial primary osteoblasts were isolated from calvarias of the WT and S89G-DMP1 mice. ALP staining showed decreased osteogenic activity in primary osteoblasts after the loss of DMP1-PG (Fig. 4A). Furthermore, the mRNA expression levels of bone formation-related proteins and matrix PGs displayed an obviously decreasing trend in the culture aggregates of the S89G-DMP1 mice compared with the WT mice (Fig. 4B). The abnormal osteogenic ability of primary osteoblasts from the S89G-DMP1 mice further confirmed the positive role of DMP1-PG in modulating cranial defect healing.

### Inflammation-related signaling pathways involved in defect healing

**Fig. 5:**
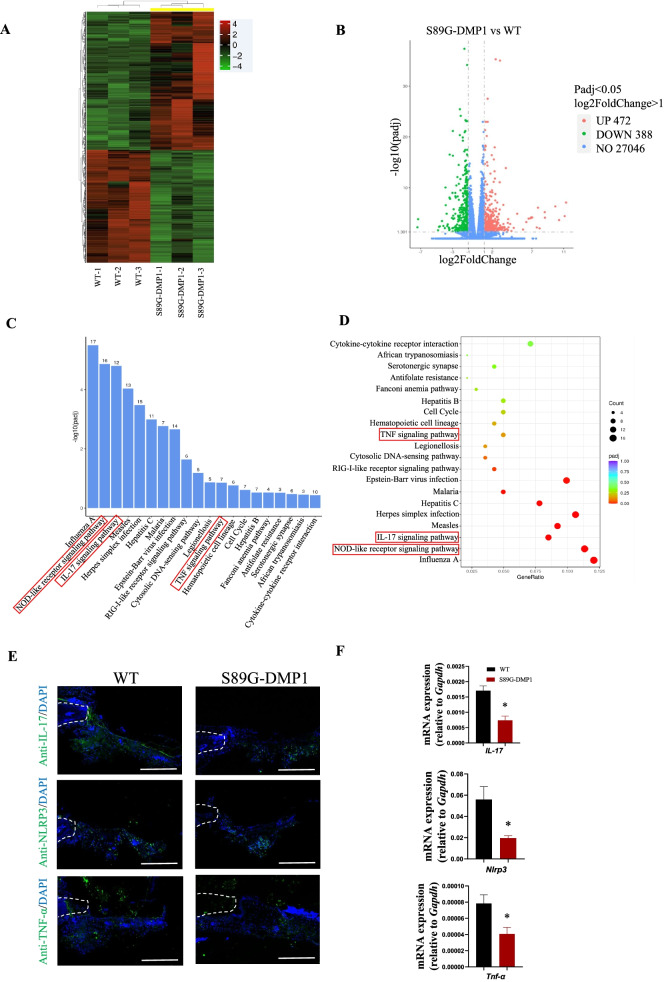
The differential transcriptional level at defect sites in the WT and S89G-DMP1 mice. **A** Heatmap of the analysis profiles of defect sites between the WT and S89G-DMP1 groups. **B** Volcano plot analysis of differential genes at defect sites in the two groups. KEGG analysis (**C**) and gene ratio analysis (**D**) displayed the downregulated signaling pathways in the S89G-DMP1 mice. **E** Weaker immunoactivity of IL-17, NLRP3 and TNF-α was observed in the cranial defects in the S89G-DMP1 mice. Scale bars = 100 µm. **F** RT‒qPCR quantification of inflammatory signaling molecules in the defect sites. *P < 0.05, *n* = 6

To detect the potential biological functions of DMP1-PG in cranial defect repair, we performed RNA sequencing to evaluate the differential genes between the two groups at day 3 post-injury. Compared to that of the control group, a total of 860 genes were involved in the process of repair in the S89G-DMP1 mice: 472 genes were upregulated and 388 genes were downregulated (Fig. 5A, B).

Kyoto Encyclopedia of Genes and Genomes (KEGG) is an integrated database for systematic analysis of gene functions [17–19]. According to the KEGG analysis, several signaling pathways, including the nucleotide-binding oligomerization domain (NOD)-like receptor signaling pathway, IL-17 signaling pathway, retinoic acid-inducible gene 1 (RIG-1)-like receptor signaling pathway and tumor necrosis factor (TNF) signaling pathway, were obviously decreased (Fig. 5C, D). Immunofluorescence staining further confirmed the abnormal inflammatory activity in the S89G-DMP1 mice (Fig. 5E). The gene expression levels of major inflammatory signaling pathways were downregulated in the sites of the S89G-DMP1 mice compared with those of the control group at day 3 post-injury (Fig. 5F). The altered signaling pathways indicated that inflammation-related molecules in the early stage might be potential mechanisms impairing subsequent osteogenesis in defect healing.

## Discussion

Tumors, congenital disease, fracture or trauma can cause cranial defects that are still challenging to heal. Following skull damage, bone regeneration begins along with hematoma formation and inflammatory cell influx. The secretion of growth factors and cytokines released by endothelial and inflammatory cells leads to the recruitment of MSCs to sites of injury and their differentiation into osteoblasts secreting mineralized matrix [20]. During the process of healing, the regenerative capacity can be affected by several bioactive factors, including cellular and extracellular signaling molecules [14, 21]. As an important group of ECM, PGs can act as a framework and regulate several signaling cascades controlling various biological processes [22]. Many studies have confirmed that PGs are widely and differentially expressed in the stages of bone development and trauma repair [23–25]. PGs are extensively involved in a variety of bone biological functions, such as maintaining the differentiation of MSCs, binding calcium and phosphate and interacting with growth factors. Breakdown of specific PGs in mice results in early onset of osteoporosis and delays fracture healing [26, 27].

DMP1-PG is a novel acidic ECM PG that is highly expressed in the bone matrix, cartilage and fracture callus. DMP1-PG deficiency could lead to abnormal craniomaxillofacial development and delayed fracture healing in long bones [16]. However, we still have limited knowledge about the special role of DMP1-PG in cranial defect repair. Compared with the endochondral ossification of long bone fracture healing, the process of cranial defect repair shows an intramembranous ossification pattern in the cranial vault, which is characterized by triggering the recruitment and differentiation of stem cells into the bone-forming cell lineage. Based on our investigations, we found that the PG form of DMP1 was highly expressed in the callus of the cranial defect area and cranial matrix at days 7 and 28. Furthermore, the mRNA expression level of *Dmp1* was upregulated continuously compared with that in the normal cranial matrix after defect. The above results indicated that DMP1-PG might function to regulate osteogenesis during the process of cranial defect repair.

In previous research, glycosylation of DMP1 was fully blocked in HEK-293 cells by the S89G substitution [28]. However, in vivo, the S89G substitution could decrease, but not fully block, the glycosylation of DMP1 in the bone matrix of S89G-DMP1 mice [11]. This finding indicates that the blocking efficiency of S89G might be incomplete in mice or that there is a backup glycosylation site for mouse DMP1 when the S89G substitution is made. To further detect the potential role of DMP1-PG in maintaining bone homeostasis during defect healing, we used S89G-DMP1 mice and control mice to establish a cranial defect model. In terms of defect repair, reduced calluses bridging the defect areas and delayed intramembranous ossification were found in the S89G-DMP1 mice compared with the WT mice. Except for the discrepancy in histological staining, the protein signal using the immunofluorescence staining technique indicated weaker osteogenic activity compared to that of the WT mice. At the gene level, the expression levels of osteogenesis and PG-related markers were also significantly decreased in the defect sites of the S89G-DMP1 mice. All of these changes revealed that the glycosylated form of DMP1 is a critical ECM PG in defect fusion.

As an important component of the bone matrix, PGs could be involved in maintaining the remodeling microenvironments of osteoblasts, and the deficiency of PGs will lead to abnormalities in bone remodeling [29]. In our studies, we found that the loss of DMP1-PG could inhibit the gene expression levels of other PGs, including *Acan*, *Bgn*, *Dcn* and *Vcan*, during the cranial repair phase, which was consistent with the changes revealed in the *Bgn*-deficient mouse bone injury model. This finding indicated that DMP1-PG could serve as a key regulator in the metabolic activity of other PGs during cranial defect healing.

Osteoblasts derived from multipotent MSCs are critical players in bone formation and repair [30]. During bone healing, osteoblasts can proliferate and deposit bone ECM components in the injury sites [31]. Several studies have demonstrated that primary osteoblast cultures can heal bone defects, which has been suggested as a potential approach for bone defect therapy [32–34] In this research, the primary osteoblasts of both groups were separated and cultured in osteogenic medium, and weakened ALP staining and decreased expression levels of osteogenic marker genes were observed in the culture aggregates of the S89G-DMP1 mice. Current studies at the cellular level indicated that the integrated and functional DMP1-PG is indispensable in maintaining the full osteogenic potency of osteoblasts, and these findings were correlated with the potential role of PGs in controlling cellular behavior and regulating tissue morphogenesis [35].

During the process of defect healing, several known signaling molecules, including the transcription factors Runx2 and Osterix, Wnt signaling, transforming growth factor β (TGF-β) superfamily signaling, Notch signaling, fibroblast growth factor (FGF) ligands, vascular endothelial growth factor (VEGF) signaling and hypoxia inducible factor-1α (Hif1α) signaling, are involved in injury site ossification [36–41]. To further probe the molecular mechanism of DMP1-PG in regulating defect healing, we performed RNA transcription analysis. The results from transcriptional sequencing indicated that several signaling pathways related to inflammatory reactions were downregulated significantly in the S89G-DMP1 mice compared with the WT mice during injury repair. NOD-like receptor signaling molecules have been demonstrated to play critical roles in maintaining the metabolic balance of bone tissues and regulating osteogenesis [42]. IL-17 signaling molecules are involved not only in the inflammatory effect but also in the process of systemic bone loss [43]. In addition, we found a decrease in the TNF signaling pathway by using transcription analysis. As important inflammatory cytokines, TNF superfamily members can induce the differentiation and activity of osteoblasts and osteoclasts and regulate bone homeostasis [44]. The ECM, in particular PGs, plays a wide-ranging role in the immune system and in wider biology [45]. Several studies have demonstrated the complex biological functions of PGs in signaling to immune system to recruit immune cells to injury sites. PGs can serve as key modulators of immune cell recruitment and positioning [46]. The decreased DMP1-PG in the cranial defect areas of S89G-DMP1 mice might result in an abnormal immune response and the release of inflammatory signaling molecules followed by the early stage of injury. Based on our data, we inferred that the impaired immune signaling pathway might be involved in the osteogenic ability of osteoblasts during defect healing in the S89G-DMP1 mice due to the breakdown of DMP1-PG.

## Conclusion

In summary, our studies provide evidence that the PG form of DMP1 can effectively facilitate cranial defect healing. Given its positive effects on bone formation and healing, DMP1-PG might also have potential roles in molecular therapy to drive bone tissue regeneration.

## Materials and methods

### Animals

All experimental procedures using animals were approved by the animal welfare committee of Qiqihaer Medical University. The generation of S89G-DMP1 mice has been described in a previous study [11]. Briefly, according to the whole genome sequence of wild-type mice (C57BL/6 J), homologous recombination arms of 6.6 kb and 8.2 kb gene sequences of all DMP1 protein glycosylation site exons were constructed. The Neo- positive sequence was knocked in 179 bp downstream of exon 6 of DMP1. After the plasmid was constructed, it was transferred into mouse embryonic stem cells by electroporation technology, and then the 3 positive clones were transplanted into the uteri of Balbc pseudopregnant mice. Male mice with 5 chimeras were crossed with female wild-type mice (C57BL/6 J) to obtain F1 generation mice. The male mice obtained by hybridization were crossed with B6.129S4-Gt (ROSA) 26-Sortm1 (FLP1) Dym/RainJ female mice to remove the Neo sequence, and the progeny mice with the Neo-removed sequence were genotyped. All mice were maintained in an SPF facility at 22 °C.

### Cranial defect model

WT mice and S89G-DMP1 mice underwent a cranial defect operation as described previously [2]. In brief, 8-week-old male WT mice and S89G-DMP1 mice were employed to construct the cranial defect model. After anesthetization with isoflurane, the overlying pericranium of the mice was removed, and a circular, full depth defect hole with a diameter of 1.2 mm was created with a turbine drill on the right parietal. Dural integrity was maintained throughout the procedure. The periosteum adjacent to the defect site was protected carefully, and then, 3–0 nylon sutures were employed to close the skin incision. Analgesics were used via intraperitoneal injection for 3 days post- injury.

### Micro-CT analysis

The WT and S89G-DMP1 mice were sacrificed at days 7, 14 and 28 post-operation. After the mice were sacrificed, the thoracic cavity was opened immediately to expose the heart, and the perfusion needle was inserted into the left ventricle to replace the general blood by using 40 g/L paraformaldehyde (PFA). The craniofacial complex was separated from mice, and the cranial defect specimens were soaked in 4% PFA for 72 h. Then, a micro-CT scan was performed using a high resolution μCT 50 instrument (SCANCO, Switzerland) with the following settings: voltage of 70 kVb, current of 200 μA and resolution of 10 μm. Mimics 13.0 software was used to reconstruct and analyze image data. For quantitative analysis, a 1.2-mm-diameter area in the center of the cranial injury site was selected as the volume of interest (VOI) [47]. Percentage healing was evaluated by micro architectural parameters, including BV/TV, Tb.N and Tb.Th, according to the protocol supported by the manufacturer of the μCT.

### Histological and immunofluorescence analyses

For histological analyses, samples from cranial bones were dissected and fixed in 40 g/L PFA for 72 h at 4 °C. The craniofacial complex was decalcified in 100 g/L ethylene diamine tetraacetic acid (EDTA) solutions (Sangon Biotech) at 4 °C for 14 days. The samples were dehydrated through a graded ethanol series, embedded in paraffin and then cut into 4 μm sections. Then, the sections were deparaffinized in xylene solutions for 20 min and rehydrated in a descending series of ethanol concentrations (1000 ml/L for 5 min, 900 ml/L for 5 min and 750 ml/L for 5 min). To assess the extent of the repair healing, we generated serial sections with hematoxylin and eosin (H&E) staining (Sangon Biotech) and toluidine blue (Sangon Biotech) staining in the center of the defect as previously reported [48].

For the immunofluorescence assay, the sections were treated with hyaluronidase for antigen retrieval and goat serum for blocking. Primary antibodies against RUNX2 (Abcam, rabbit), ALP (Abcam, rabbit), IL-17 (Proteintech, rabbit), TNF-α (Proteintech, mouse), NLRP3 (Proteintech, rabbit) and DMP1-N.9B6.3 (a gift from Dr. Chunlin Qin, Baylor College of Dentistry, rabbit) were used at a dilution of 1:500. Then the defect sections were incubated with Alexa Fluor 488 and 546 (1:500; Invitrogen, donkey) for 1 h at 37 °C and DAPI (Sigma-Aldrich) was used as a counterstain for 20 min in the dark. All the primary antibodies were packaged separately and stored at -20 °C to maintain the activity.

### Real-time quantitative polymerase chain reaction (RT‒qPCR)

Total RNA was isolated from cranial bone defect areas at the indicated time points as described earlier [49]. In brief, the reconstructed tissues in the defect areas were isolated carefully and then placed in 1000 μL of TRIzol reagent (Invitrogen) for homogenization according to the manufacturer’s protocol. A Transcriptor First Strand cDNA Synthesis Kit (Roche) was used to synthesize the cDNA. A Light Cycler 96 PCR system (Roche) was employed to detect and evaluate the differences in target genes between the two groups, and reactions of samples were run in triplicate. Relative expression was determined in relation to the housekeeping gene *Gapdh*. The sequences of the primers are listed in Supplementary Table 1.

### Western blotting

Total proteins were separated from the skulls of WT mice and S89G-DMP1 mice to detect the expression level of DMP1-PG. A bicinchoninic acid protein assay kit (Sigma-Aldrich) was used to detect the protein concentration. Anti-DMP1-N.9B6.3 (a gift from Dr. Chunlin Qin, Baylor College of Dentistry, rabbit) was employed at a dilution and anti-β-actin (Sigma-Aldrich, mouse) antibody was used to probe each sample.

### Cell isolation and culture

Mouse cranial primary osteoblasts were isolated from the calvarias of 3-day-old mice in both groups. Briefly, after adequate anesthesia, the skin around the skull was removed carefully. The skulls were gently incubated with 2.5 g/L trypsin (Gibco) for 10 min and collagenase (Sigma-Aldrich) for 20 min at 37 °C. Then, centrifugation at 1200 r/min for 5 min was used to separate and harvest the cells. The skull cells were cultured in 12-well dishes consisting of 100 ml/L fetal bovine serum (Excell), α-MEM culture medium (Sigma-Aldrich) and 10 mg/L penicillin/streptomycin (Sigma-Aldrich). Medium changes were carried out every 2 days, and when the cell density reached 80%, the cells were trypsinized and passaged at a ratio of 1:2. For osteoblastic induction evaluation, the cells of p3 generation were cultured in osteoblastic medium containing 50 μg/mL ascorbic acid (Sigma-Aldrich), 10 nM dexamethasone (Sigma-Aldrich) and 100 mM β-glycerophosphate (Sigma-Aldrich) for 21 days. For ALP staining, cells were cultured on 24-well plates and stained with an ALP kit (Sangon Biotech) according to the manufacturer's instructions at day 14 after osteogenic induction.

### RNA sequencing and data analysis

Total RNA was isolated from defect areas of the WT mice and S89G-DMP1 mice at day 7 post-injury. Before sequencing, the RNA purity was evaluated by using a NanoVue (GE), and RNA integrity was assessed using the Agilent 2200 Tape Station (Agilent Technologies). The RNA transcription analysis was performed at Novogene Co., Ltd., Beijing. All the differentially expressed genes were used for heatmap analysis. KEGG analysis was employed to detect the abnormal signaling pathways. The differential genes were annotated in KEGG database entries, the number of differential genes in each KEGG entry was calculated, and then, a hypergeometric test was used for statistics. The annotated results were compared with the genomic background to screen significantly enriched KEGG items in the differential genes. The calculated *p* value was corrected through multiple hypothesis testing. ClusterProfiler (3.8.1) software was used for KEGG enrichment analysis, and padj < 0.05 was the threshold for KEGG item significant enrichment of differentially expressed genes.

### Statistical analysis

GraphPad Prism 7 software was applied to compare the differences between two groups, and Student’s *t* test was performed for data analysis. Two-way ANOVA was applied for the comparison of three groups. The results are presented as the mean ± SEM, and *p* < 0.05 was deemed statistically significant.

## Supplementary Information

**Additional file 1:** **SupplementaryTable 1.**

**Additional file 2.** 

**Additional file 3.** 

## Data Availability

The datasets used and/or analyzed in this study can be requested from the corresponding author. The high-throughput RNA-seq datasets are available in the Sequence Read Archive (SRA) database of NCBI, and the accession code to the SRA database is PRJNA810771.
