# Supplementary material for: The role of proteoglycan form of DMP1 in cranial repair
Source: BMC Mol Cell Biol. 2022 Sep 30;23:43. doi: 10.1186/s12860-022-00443-4 (PMC9524138; doi:10.1186/s12860-022-00443-4)
Supplement: Supplementary file 1 — Additional file 1: SupplementaryTable 1. [file 12860_2022_443_MOESM1_ESM.doc]

Supplementary Table 1

| Genes Sequence |
| --- |
| *Dmp1*-F 5'-AGTGAGTCATCAGAAGAAAGTCAAGC-3'  *Dmp1*-R 5'-CTATACTGGCCTCTGTCGTAGCC-3'  *Opn*-F 5'-GATCAGGACAACAACGGAAAGG-3'  *Opn*-R 5'-GCTGGCTTTGGAACTTGCTT-3'  *Bsp*-F 5'-AGGACTGCCGAAAGGAAGGTTA-3'  *Bsp*-R 5'-AGTAGCGTGGCCGGTACTTAAA-3'  *Ocn*-F 5'-GAGGACCATCTTTCTGCTCACTCT-3'  *Ocn*-R 5'-TTATTGCCCTCCTGCTTGGA-3'  *Alp*-F 5'-GATCATTCCCACGTTTTCACATT-3'  *Alp*-R 5'-TTCACCGTCCACCACCTTGT-3'  *Opg*-F 5'-CCAAGAGCCCAGTGTTTCTT-3'  *Opg*-R 5'-CCAAGCCAGCCATTGTTAAT-3'  *Runx2*-F 5'-GCACAAACATGGCCAGATTCA-3'  *Runx2*-R 5'-AAGCCATGGTGCCCGTTAG-3'  *Acan-*F 5'-CGCCACTTTCATGACCGAGA-3'  *Acan-*R 5'-TCATTCAGACCGATCCACTGGTAG-3'  *Bgn-*F 5'-CCTGAGACCCTGAACGAAC-3'  *Bgn-*R 5'-GTGACCTAAGCCCAACCTGT-3'  *Dcn-*F 5'-TGAGCTTCAACAGCATCACC-3'  *Dcn*-R 5'-AAGTCATTTTGCCCAACTGC-3'  *Vcan-*F 5'-TGAGCTTCAACAGCATCACC-3'  *Vcan-*R 5'-ATAGAATTGTCCTTTGCGGATGAGG-3'  *Nlrp3-*F 5'-ATGCTG CTTCGA CAT CTC CT-3'  *Nlrp3-*R 5'-AAC CAA TGCGAG ATC CTG AC-3'  *IL-17*-F 5'-GAAGGCCCTCAGACTACCTCAA-3'  *IL-17-R* 5'-TCATGTGGTGGTCCAGCTTTC-3'  *Tnf-α-F* 5'-GGCAGGTCTACTTTGGAGTCAT-3'  *Tnf-α-R* 5'-CAGAGTAAAGGGGTCAGAGTGG-3' |
