# Supplementary figures and images for: The role of proteoglycan form of DMP1 in cranial repair
Source: BMC Mol Cell Biol. 2022 Sep 30;23:43. doi: 10.1186/s12860-022-00443-4 (PMC9524138; doi:10.1186/s12860-022-00443-4)

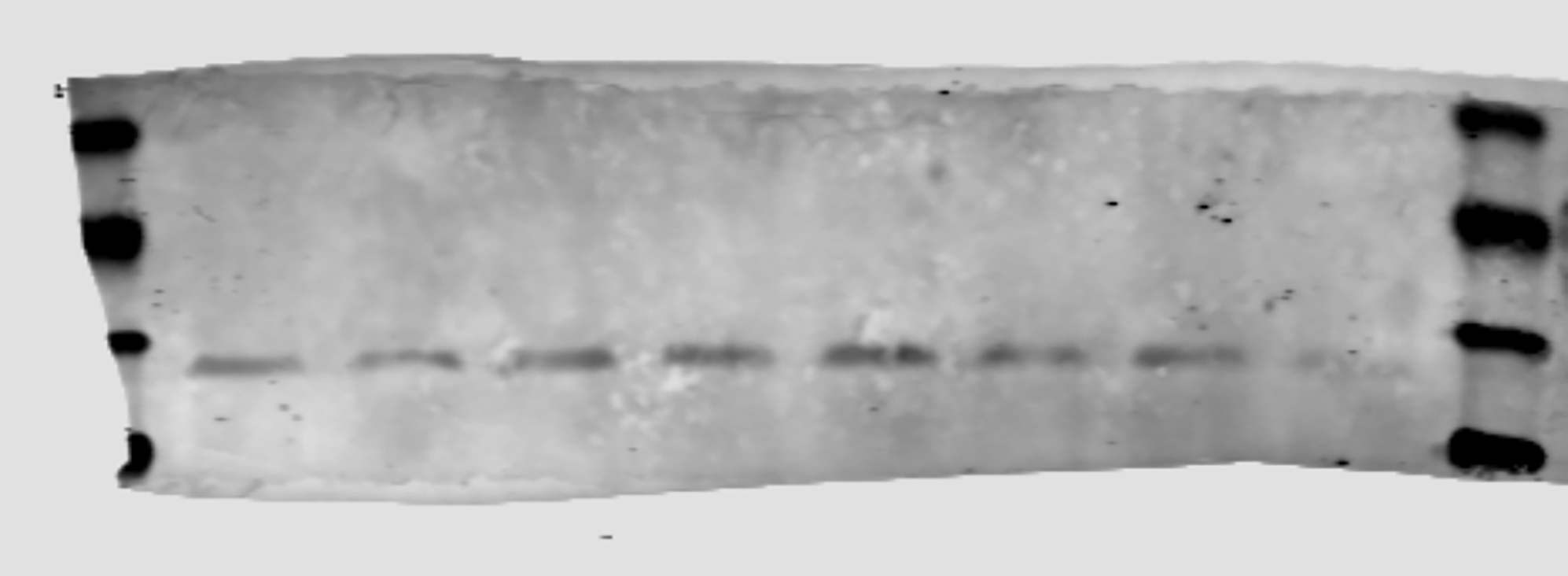

Supplement: Supplementary file 2 — Additional file 2. [file 12860_2022_443_MOESM2_ESM.tif]

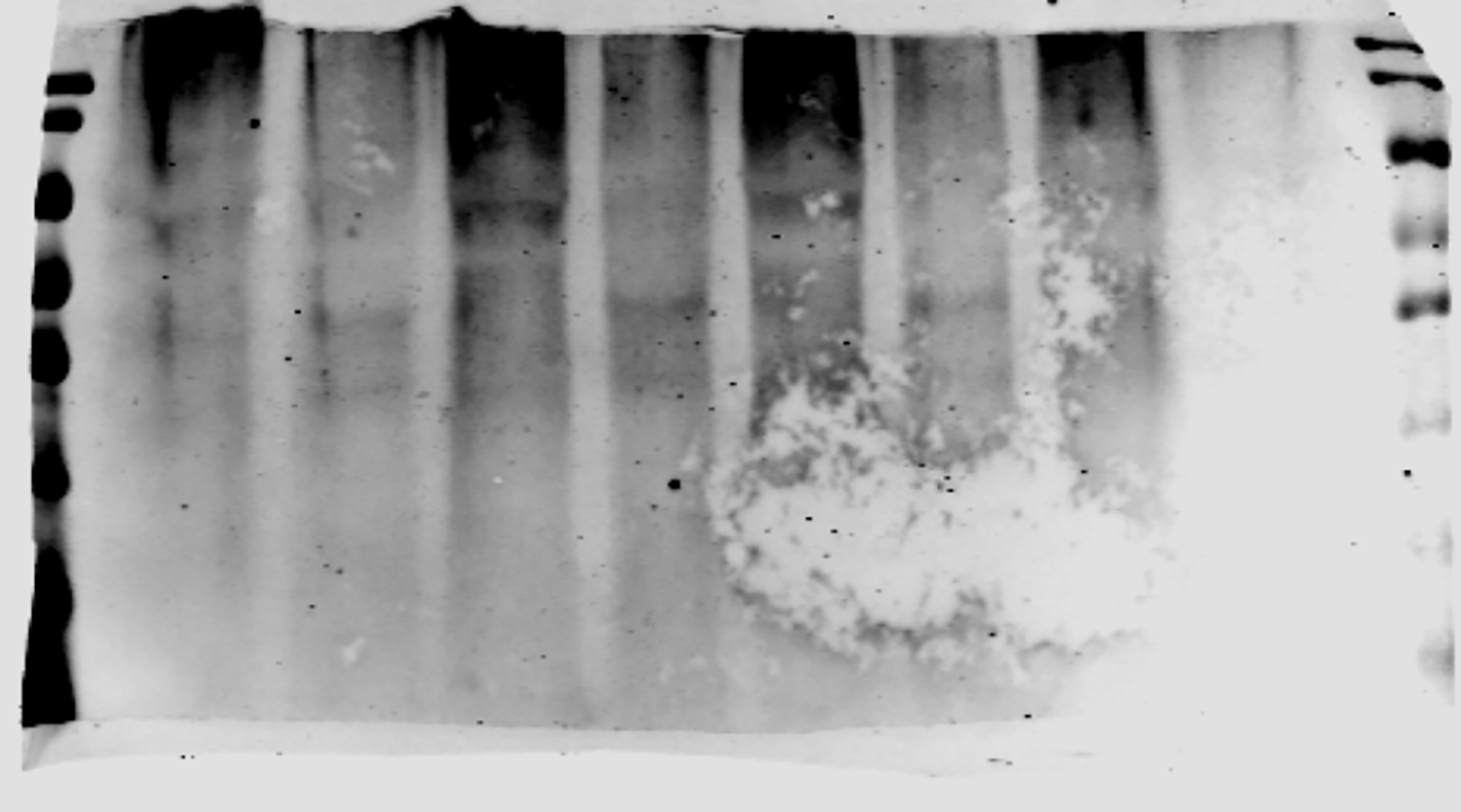

Supplement: Supplementary file 3 — Additional file 3. [file 12860_2022_443_MOESM3_ESM.tif]
